# Protocol for a Pilot Quasi‐Experimental Study on the Effect of Patient‐Centered Education on HIV Virological Outcomes Among Adult People Live With HIV‐HBV Co‐Infection in North‐West Ethiopia: Protocol for a Pilot Quasi‐Experimental Study

**DOI:** 10.1002/hsr2.73167

**Published:** 2026-08-31

**Authors:** Mequanente Dagnaw, Destaw Fetene Teshome, Tilahun Bizuayehu Demass, Abebaw Gebyehu Worku

**Affiliations:** ^1^ Department of Epidemiology and Biostatistics, Institute of Public Health University of Gondar Gondar Ethiopia; ^2^ Department of Medical Biotechnology, Institute of Biotechnology University of Gondar Gondar Ethiopia; ^3^ Department of Internal Medicine, College of Medicine and Health Sciences University of Gondar Gondar Ethiopia; ^4^ Reproductive Health Department, Institute of Public Health, College of Medicine and Health Sciences University of Gondar Gondar Ethiopia

**Keywords:** adult people live with HIV, Ethiopia, health education, virological suppression

## Abstract

**Background:**

Human immunodeficiency virus (HIV) remains a major public health challenge in sub‐Saharan Africa, including Ethiopia. Adults living with HIV are at increased risk of liver‐related complications, poor treatment outcomes, and suboptimal virological control, particularly when adherence to antiretroviral therapy (ART) is inadequate. Patient‐centered education has emerged as a promising strategy for improving treatment adherence, self‐management, and health outcomes among individuals with chronic diseases. However, evidence on the effect of patient‐centered education on HIV virological outcomes among adults living with HIV remains limited, particularly in resource‐constrained settings such as North‐West Ethiopia. Furthermore, limited evidence exists regarding the feasibility and acceptability of structured educational interventions tailored to this vulnerable population. This pilot quasi‐experimental study therefore aims to assess the effect of a patient‐centered education intervention on HIV virological outcomes among adults living with HIV in North‐West Ethiopia.

**Objective:**

To evaluate the feasibility and preliminary effectiveness of a patient‐centered educational intervention on HIV virological outcomes, medication adherence, and HIV‐related knowledge among 120 adult people living with HIV–HBV co‐infection receiving ART in North‐West Ethiopia.

**Methods and Analysis:**

This pilot quasi‐experimental study will employ non‐randomized intervention and comparison groups with baseline and 6‐month follow‐up assessments. A total of 120 participants (60 per group) will be enrolled from selected hospitals in North‐West Ethiopia. Participants in the intervention group will receive a structured patient‐centered education program integrated into routine ART services, while the comparison group will continue receiving standard care. Quantitative data will be entered into EpiData version 4.6 and analyzed using STATA version 18. Descriptive statistics will summarize baseline characteristics. Baseline differences between groups will be assessed using chi‐square tests for categorical variables and independent t‐tests or non‐parametric equivalents for continuous variables. The primary outcome will be the change in log‐transformed HIV viral load (log_10_ copies/mL) from baseline to the end of the 6‐month follow‐up period. The intervention effect will be evaluated using paired t‐tests, difference‐in‐differences analysis, and multivariable linear regression while adjusting for potential confounders, including age, sex, educational status, baseline viral load, duration of ART, and medication adherence. Virological suppression, defined as HIV viral load < 1000 copies/mL, will be assessed as a secondary clinical outcome. Secondary outcomes will include medication adherence, HIV‐HBV‐related knowledge, self‐management practices, and retention in care. Statistical significance will be declared at *p* < 0.05. A qualitative study will be conducted at the end of the intervention period to assess the feasibility, acceptability, and implementation potential of the intervention. Qualitative data will be collected through focus group discussions with adult people live with HIV, in‐depth interviews with healthcare providers, and key informant interviews with hospital leaders and program managers. Audio‐recorded interviews will be transcribed verbatim and analyzed using directed thematic analysis with MAXQDA version 20.

**International Trial Registration Number:**

The intervention protocol was registered on 15 November 2025 with the Pan African Clinical Trials Registry under identification number PACTR202512648125852.

**Abbreviations**
AASLD: American Association for the Study of Liver Disease
ADEs: adverse drug events
AIDS: acquired immune deficiency syndrome
ALT: alanine aminotransferase
AOR: adjusted odds ratio
ART: antiretroviral therapy
CHIV: chronic hepatitis B
CI: confidence intervals
CONSORT: consolidated standards of reporting trials
DAAs: direct‐acting antivirals
DNA: deoxyribose nucleic acid
EFA: exploratory factor analysis
ETV: entecavir
FGD: focus group discussion
GFI: goodness‐of‐fit index
HAART: highly active antiretroviral therapy and HBeAb Hepatitis B(e) antibody
HBeAg: hepatitis B e‐antigen
HBsAg: hepatitis B surface antigen
HCC: hepatocellular carcinoma
HCPs: health care providers
HIV: human immunodeficiency virus
HRQoL: health‐related quality of life
IDI: in‐depth interview
IFN‐a: interferon‐alpha
IQR: interquartile range
IRB: Institutional Review Board
IV: intra veinous
JBI: Joana Brig's Institute
KIIs: key informant
LMICs: low‐and middle‐income countries
MAXQDA: MAX qualitative data analysis
MCS: mental component summary
MeSH: medical subject headings
MH: mental health
MOPs: manual of operating procedures
NAs: nucleotide analogs
NUCs: nucleotide analogs,
OPD: out patient department
PCPs: primary care providers
PCS: physical component summary
PF: physical functioning
PLHIV: people living with HIV
SD: standard deviations
SF: social functioning
SSA: Sub‐Saharan Africa
SVR: sustain virological response
TDF: tenofovir disoproxil fumarate
WHO: World Health Organization.

## 1 Introduction

Human immunodeficiency virus (HIV) infection remains one of the major global public health concerns, particularly in sub‐Saharan Africa where the burden of disease is disproportionately high [1]. Hepatitis B Virus (HBV) infection is also highly endemic in the region, and co‐infection with HIV is common because both viruses share similar routes of transmission, including sexual contact, blood exposure, and mother‐to‐child transmission [2]. HIV has become an important clinical and public health issue because it accelerates disease progression, complicates treatment outcomes, and increases morbidity and mortality among affected individuals [3].

Globally, approximately 38 million people are living with HIV, and nearly 7%–10% of them are co‐infected with chronic HBV infection [2]. In Ethiopia, studies have reported varying prevalence rates of HIV ranging from 5% to 20% among people receiving antiretroviral therapy (ART) services [4, 5]. The presence of HBV among people living with HIV contributes to rapid liver disease progression, hepatocellular carcinoma, poor immune recovery, and increased risk of treatment failure [6, 7].

The primary goal of ART is achieving sustained HIV virological suppression, which improves quality of life, decreases HIV transmission, and reduces mortality [8]. However, maintaining viral suppression among HIV‐HBV co‐infected patients remains challenging because of poor medication adherence, inadequate counseling, psychosocial barriers, low health literacy, and co‐morbid conditions [9]. Studies from Ethiopia and other African countries indicate that suboptimal adherence remains one of the strongest predictors of virological failure among ART users [10, 11].

Patient‐centered education is increasingly recognized as an effective strategy to improve adherence and treatment outcomes among patients with chronic illnesses. Patient‐centered educational interventions involve individualized counseling, shared decision‐making, behavioral support, and tailored health information that addresses patients' specific needs and challenges [12]. Evidence suggests that educational interventions can improve ART adherence, retention in care, self‐management practices, and viral suppression among people living with HIV [13, 14]. Despite these benefits, limited evidence exists regarding the effectiveness of patient‐centered education specifically among HIV‐HBV co‐infected adults in resource‐limited settings such as Ethiopia.

North‐West Ethiopia is among the regions with a substantial burden of HIV infection and expanding ART services. However, there is inadequate evidence regarding intervention‐based approaches aimed at improving virological suppression among HIV‐HBV co‐infected individuals in the region. Therefore, this pilot quasi‐experimental study seeks to evaluate the effect of patient‐centered education on HIV virological suppression among adult people living with HIV in North‐West Ethiopia. The study findings may provide preliminary evidence for designing larger intervention studies and integrating patient‐centered educational strategies into routine HIV care programs.

Several studies have documented that HIV negatively affects treatment outcomes and overall health status among people living with HIV. HIV infection accelerates HBV replication and liver disease progression, while HBV co‐infection contributes to increased hepatotoxicity and impaired immune recovery during ART [6, 7]. Research has also shown that co‐infected individuals are more likely to experience poor HIV virological suppression compared with HIV mono‐infected patients [15].

In Ethiopia, evidence indicates that hepatitis co‐infection is associated with lower rates of viral suppression and higher levels of treatment complications among ART users [11]. Poor adherence to ART medications has consistently been identified as a major contributor to virological failure [10]. Studies conducted in sub‐Saharan Africa revealed that patients who receive regular counseling and educational support demonstrate significantly better adherence and viral suppression outcomes than those receiving routine care alone [13, 14].

Patient‐centered education has been widely recommended as a key strategy for chronic disease management because it improves patients' knowledge, motivation, communication, and participation in care [12]. In HIV care settings, individualized counseling and adherence‐focused interventions have shown positive effects on treatment retention and medication adherence [9]. Furthermore, integrated care approaches for HIV and HBV management have demonstrated feasibility and acceptability in low‐resource settings [16].

The World Health Organization and Ethiopian national HIV treatment guidelines emphasize the importance of adherence counseling, continuous patient education, and psychosocial support as essential components of comprehensive HIV care [2, 17]. Nevertheless, implementation of structured patient‐centered educational interventions specifically targeting HIV‐HBV co‐infected individuals remains limited in many healthcare settings.

Although evidence exists regarding the epidemiology and clinical outcomes of HIV‐HBV co‐infection, major gaps remain concerning effective behavioral and educational interventions for improving virological suppression among co‐infected patients. Most previous studies conducted in Ethiopia focused on prevalence, associated factors, and clinical characteristics of HIV rather than intervention‐based strategies [4, 5].

There is limited evidence on whether patient‐centered education can significantly improve HIV virological suppression among adults living with HIV in resource‐constrained settings. Existing studies on adherence interventions mainly target general HIV populations without specifically addressing the unique challenges faced by HIV‐HBV co‐infected individuals, such as liver‐related complications, treatment burden, stigma, and complex medication management [15].

Additionally, little is known about the feasibility and acceptability of implementing structured patient‐centered educational interventions within routine ART clinics in North‐West Ethiopia. Health facilities in the region often experience shortages of trained healthcare providers, limited counseling resources, and high patient loads, which may affect the delivery and sustainability of such interventions [17].

Furthermore, evidence is scarce regarding the extent to which culturally tailored educational interventions can influence patients' adherence behavior, knowledge, self‐management practices, and long‐term virological outcomes among HIV‐HBV co‐infected populations in Ethiopia. Therefore, this pilot quasi‐experimental study is intended to address these knowledge gaps and generate preliminary evidence to inform future large‐scale intervention studies and healthcare policy development.

## 2 Methods

### 2.1 Study Objective

#### 2.1.1 Primary Objective

The primary objective of this study is to evaluate the effect of a patient‐centered education program on HIV virological outcomes among adults living with HIV‐HBV co‐infection. The corresponding primary outcome will be the change in plasma HIV RNA viral load, measured as log‐transformed HIV viral load (log_10_ copies/mL) from baseline to the end of the 6‐month follow‐up period. This outcome will be assessed using plasma HIV RNA viral load testing to quantify changes in virological response over time. In addition, HIV virological suppression, defined as a plasma HIV viral load of < 1000 copies/mL, will be evaluated as a secondary clinical outcome at the end of follow‐up.

#### 2.1.2 Secondary Objectives

To assess the effect of the patient‐centered education intervention on medication adherence to ART and HBV‐active medications among adults living with HIV‐HBV co‐infection.

The corresponding secondary outcome will be medication adherence, measured using the context‐adapted Hill‐Bone Medication Adherence Scale (HBMAS) and analyzed using both continuous adherence scores and categorical adherence levels (good vs. poor adherence).

To evaluate changes in participants' knowledge and awareness regarding HIV following the patient‐centered education intervention.

The corresponding secondary outcome will be the HIV‐HBV‐related knowledge score, measured using a structured questionnaire and reported as a composite score, with higher scores indicating better knowledge and awareness.

To assess the effect of the patient‐centered education intervention on retention in HIV care among adults living with HIV‐HBV co‐infection.

The corresponding secondary outcome will be retention in care, measured by participants' attendance at scheduled clinic visits, continuity of follow‐up care, and adherence to routine clinical monitoring throughout the 6‐month study period.

#### 2.1.3 Exploratory Objectives

To explore factors associated with HIV virological suppression among adults co‐infected with HIV and HBV, including sociodemographic, clinical, and behavioral characteristics.

To explore the relationship between changes in HIV‐HBV‐related knowledge, ART adherence, and HIV virological suppression following the intervention.

To assess the acceptability and feasibility of implementing the patient‐centered education program in routine HIV care settings.

### 2.2 Research Question

This study seeks to address the following research questions: What is the effect of a patient‐centered education intervention on HIV virological outcomes among adults living with HIV in North‐West Ethiopia? To what extent does the intervention improve participants' knowledge of HIV‐HBV co‐infection, self‐management practices, medication adherence, and retention in care? In addition, the study will explore the individual, behavioral, clinical, and health system factors that may influence the implementation and effectiveness of the intervention. Furthermore, the study will assess the feasibility, acceptability, and potential integration of the patient‐centered education intervention into routine ART service delivery.

### 2.3 Null Hypothesis

The null hypothesis of this pilot quasi‐experimental study states that the patient‐centered education intervention will have no statistically significant effect on HIV virological outcomes among adults living with HIV in North‐West Ethiopia compared with standard care. Specifically, it assumes that there will be no significant difference between the intervention and comparison groups in the change in HIV viral load over the study period. Furthermore, the intervention is hypothesized to produce no significant improvement in secondary outcomes, including medication adherence, self‐management practices, retention in care, and patient knowledge regarding HIV‐HBV co‐infection. In addition, the intervention is assumed to demonstrate no significant difference in feasibility, acceptability, or potential for integration into routine ART service delivery compared with standard practice.

### 2.4 Alternate Hypothesis

The alternative hypothesis of this pilot quasi‐experimental study is that, compared with standard care, the patient‐centered education intervention will result in a statistically significant improvement in HIV virological outcomes among adults living with HIV in North‐West Ethiopia. Specifically, participants receiving the intervention are expected to demonstrate greater reductions in HIV viral load over the study period compared with those receiving standard care. Furthermore, the intervention is hypothesized to significantly improve secondary outcomes, including medication adherence, self‐management practices, retention in care, and patient knowledge regarding HIV‐HBV co‐infection. Additionally, the intervention is expected to be feasible, acceptable, and suitable for integration into routine ART service delivery, thereby demonstrating potential for broader implementation in similar healthcare settings.

### 2.5 Trial Design

To assess the effect of a patient‐centered education intervention on HIV‐related clinical outcomes among adults living with HIV–HBV co‐infection in North‐West Ethiopia, this study will employ a pilot quasi‐experimental pre–post design with non‐randomized intervention and comparison groups. Participants receiving care at the comparison site will continue to receive routine standard HIV care, whereas participants at the intervention site will receive a structured patient‐centered education program integrated into routine ART services. The intervention effect will be evaluated by comparing changes in outcomes over time between the two groups using a difference‐in‐differences approach. The primary outcome will be assessed as a continuous measure, specifically the change in log‐transformed HIV viral load (virological response), while secondary outcomes will include medication adherence, self‐management practices, retention in care, and disease‐related knowledge. Outcome measurements will be collected at two time points: baseline (before intervention implementation) and endline (6 months after intervention initiation). Participant recruitment and baseline assessment will commence in December 2025, followed by intervention implementation and follow‐up from January to June 2026, with end‐line data collection completed in June 2026.

### 2.6 Study Participant

#### 2.6.1 Inclusion Criteria

Eligible participants will be adult people living with HIV aged 18 years or older who are receiving ART, have been on routine follow‐up for at least 6 months, have poor virological outcome (HIV viral load ≥ 1000 copies/mL), receive HIV care at the selected study sites, and are willing to participate in both baseline and endline assessments.

#### 2.6.2 Exclusion Criteria

Patients will be excluded if they have been on ART for less than 6 months, are unable to communicate effectively, have severe hearing impairment, are critically ill, have advanced immunosuppression (CD4 count < 200 cells/mm^3^), or have active tuberculosis, hepatitis C virus (HCV) infection, or active syphilis requiring intensive medical management, as these conditions may confound the study outcomes.

### 2.7 Sample Size Determination

The sample size for this pilot quasi‐experimental study was determined based on the primary outcome, treated as a continuous variable (such as reduction in HIV viral load, adherence score, or knowledge score). Since measurements will be taken at baseline and endline in both intervention and comparison groups, the intervention effect will be evaluated by comparing mean changes in outcome between groups using a paired *t*‐test and a difference‐in‐differences approach [18].

The required sample size was calculated using the standard formula for comparing mean differences between two independent groups [19, 20].

The sample size for this study was determined using the formula for comparing two means: n = 2σ^2^(Z_a/2_ + Z_β_)^2^/Δ^2^

where (n) represents the required sample size per group, Z_a/2_ is the critical value corresponding to the desired significance level, (Z_β_) is the critical value corresponding to the desired statistical power, σ is the standard deviation of the change score, (σ^2^) is the variance of the change score, and Δ is the expected mean difference between the intervention and comparison groups. For this study, a significance level of 5% (α = 0.05) was assumed, corresponding to a critical value of (Z_a/2_ = 1.96), while a statistical power of 80% was used, corresponding to (Z_β_ = 0.84). The standard deviation of the change score was assumed to be 15, and the expected mean difference between the two groups was 8.4. Substituting these values into the formula gave; n = 2(15)^2^(1.96 + 0.84)^2^/8.4^2^

The variance was first calculated as (15^2^ = 225), and the sum of the Z‐values was (1.96 + 0.84 = 2.80), which when squared gave 7.84. Multiplying the numerator resulted in (2 × 225 × 7.84 = 3528), while the denominator was calculated as (8.4^2^ = 70.56). Dividing the numerator by the denominator gave (n = 3528/70.56 = 49.99), which was rounded up to 50 participants per group. To account for an anticipated 20% loss to follow‐up, the sample size was adjusted using the formula (n _adjusted_ = n/1–r), where (r) represents the expected attrition rate. Substituting the values yielded (n _adjusted_ = 50/0.80 = 62.5). For operational feasibility and balanced allocation, this was rounded to 60 participants per group. Therefore, the final sample size for the study was 120 participants, with 60 participants in each group.

Thus, a total of 120 HIV–HBV co‐infected participants will be enrolled, with 60 participants in the intervention group and 60 participants in the comparison group. This sample size is also adequate for evaluating feasibility outcomes, including recruitment, retention, intervention acceptability, and preliminary effectiveness in this pilot study [21, 22].

### 2.8 Sampling Method and Subject Recruitment

Because this is a pilot quasi‐experimental study, participants will not be randomly allocated to intervention and comparison groups. Instead, the selected hospitals will be purposively assigned to either the intervention or comparison group based on service capacity, logistical feasibility, and the need to minimize contamination between study arms. The University of Gondar Comprehensive Specialized Hospital will serve as the intervention site, where participants will receive the patient‐centered education intervention integrated into routine ART services, while Felege Hiwot Comprehensive Specialized Hospital will serve as the comparison site, where participants will continue receiving standard HIV care.

Within each study site, eligible participants will be selected using simple random sampling. After obtaining the necessary institutional permissions, a sampling frame consisting of all eligible adults living with HIV–HBV co‐infection receiving ART will be obtained from the respective hospitals. Each eligible participant will be assigned a unique identification number, and participants will be selected using a computer‐generated random number method until the required sample size for each study arm is reached.

Blinding of participants and intervention providers will not be feasible because of the nature of the educational intervention. Therefore, this study will be conducted as an open‐label trial. To minimize potential bias, standardized intervention delivery procedures, uniform data collection protocols, and structured outcome assessment tools will be used across both study sites. In addition, primary outcome assessments will rely primarily on objective clinical measures, including laboratory‐confirmed HIV viral load results and routinely documented medical records, whenever possible. Although the principal investigator and data analyst will be aware of group allocation, outcome assessment and data verification procedures will be standardized to reduce measurement and analytical bias.

### 2.9 Description of Intervention Package

#### 2.9.1 Patient‐Centered Health Education Intervention

The intervention selected for this study is a patient‐centered health education intervention designed to improve HIV‐related clinical outcomes among adults living with HIV receiving ART. The intervention package consists of structured patient‐centered health education, medication adherence counseling, behavioral counseling, and self‐management support integrated into routine ART services. The primary goals of the intervention are to improve HIV virological outcomes, enhance medication adherence, strengthen self‐management practices, increase disease‐related knowledge, promote healthy behavioral changes, and improve retention in care.

Following participant enrollment, six healthcare professionals from the selected hospitals, including nurses, pharmacists, and clinicians involved in HIV care, will undergo a 3‐day training program. The training will focus on patient‐centered communication, HIV management, medication adherence counseling, behavioral change strategies, and delivery of standardized health education materials to ensure consistent intervention implementation.

Participants in the intervention group will receive structured education sessions over a 6‐month period during routine ART clinic visits. The initial session will last approximately 40–60 min and will cover general information about HIV‐HBV co‐infection, treatment goals, medication adherence, viral load monitoring, risk reduction strategies, and self‐management practices. Subsequent follow‐up sessions will be conducted monthly, with each session lasting approximately 20–30 min, to reinforce key messages, address barriers to adherence, provide individualized counseling, and support behavioral change.

Educational sessions will be delivered using standardized counseling guides, visual educational materials, and interactive discussions tailored to participants' needs, literacy levels, and clinical circumstances. Family members or treatment supporters may be involved in selected sessions, where appropriate, to strengthen adherence support. A detailed description of the intervention components, delivery procedures, and educational content will be provided following completion of the pilot study.

#### 2.9.2 Behavioral Counseling Intervention

Healthcare professionals (HCPs) will deliver the patient‐centered education intervention using the Health Belief Model (HBM) as the guiding behavioral framework. The intervention will address the core HBM constructs, including perceived susceptibility, perceived severity, perceived benefits, perceived barriers, self‐efficacy, and cues to action, to promote positive health behaviors among adults living with HIV‐HBV co‐infection.

Educational sessions will focus on improving participants' knowledge and understanding of HIV‐HBV co‐infection, including disease transmission, prevention, treatment, and long‐term management. Participants will receive information on the modes of transmission of HIV and HBV, including transmission through unprotected sexual contact, blood exposure, needle sharing, and mother‐to‐child transmission, as well as clarification of common misconceptions regarding non‐transmission through casual contact such as hugging, handshaking, sharing food utensils, or being near someone who coughs or sneezes.

The intervention will also address the chronic nature of HIV and HBV infection, the potential for disease progression and complications in the absence of treatment, and the increased risk of opportunistic infections, liver disease, cirrhosis, and hepatocellular carcinoma among individuals with HIV‐HBV co‐infection. Healthcare professionals will emphasize the benefits of early diagnosis, lifelong adherence to ART and HBV‐active medications, regular clinical follow‐up, and routine laboratory monitoring, including viral load assessment and liver function evaluation.

In addition, the educational intervention will address stigma, psychosocial barriers, and misconceptions that may negatively affect treatment adherence and healthcare‐seeking behavior. Counseling sessions will aim to strengthen patients' confidence in managing their condition, improve medication adherence, encourage retention in care, and promote self‐management practices through personalized goal setting, problem‐solving strategies, and ongoing motivational support.

#### 2.9.3 Medication Adherence Counseling

Participants will receive structured education and counseling from trained healthcare professionals on the importance of taking medications as prescribed, the potential consequences of poor medication adherence, and the clinical benefits of maintaining optimal adherence to ART and HBV‐active medications. The counseling will emphasize how consistent medication use contributes to improved virological outcomes, reduced risk of disease progression, prevention of drug resistance, and better overall health outcomes.

### 2.10 Outcome Measures

#### 2.10.1 Primary Outcome

HIV viral load will be measured at baseline and at the end of the 6‐month follow‐up period in both the intervention and comparison groups. The primary outcome will be assessed as the change in log‐transformed HIV viral load (log_10_ copies/mL) from baseline to endline, enabling quantification of the magnitude of virological response over time. The intervention effect will be evaluated by comparing the mean change in log‐transformed viral load between the intervention and comparison groups using a pre–post analytical framework.

In addition, virological suppression (VS) will be evaluated as an important clinical outcome and will be defined as an HIV viral load of < 1000 copies/mL, based on the most recent viral load measurement obtained within 30 days before or after the scheduled 6‐month follow‐up assessment. Assessments will be conducted at equivalent time points in both groups to ensure a comparable observation window. Data on viral load will be obtained from patient medical records and laboratory viral load testing reports.

#### 2.10.2 Secondary Outcomes

##### 2.10.2.1 Medication Adherence

Medication adherence will be assessed at baseline and at the scheduled 6‐month follow‐up visit in both the intervention and comparison groups using a context‐adapted version of the Hill‐Bone Medication Adherence Scale (HBMAS) [23]. Medication adherence will be evaluated over the same 6‐month post‐enrollment follow‐up period used for assessment of the primary outcome, ensuring a comparable observation window across study arms.

The HBMAS is a validated self‐report instrument designed to assess adherence behaviors among individuals receiving long‐term therapy and has been adapted to capture adherence to ART and HBV‐active medications among adults living with HIV or HIV–HBV co‐infection [23, 24]. The adapted HBMAS assesses three domains of adherence: medication‐taking behavior, appointment keeping, and prescription refilling. Responses are recorded using a Likert‐type scale, and item scores are summed to generate a total adherence score, with higher scores indicating poorer adherence.

##### 2.10.2.2 Knowledge Change

Knowledge regarding HIV–HBV co‐infection will be assessed using a structured questionnaire administered at baseline and at the end of the 6‐month follow‐up period in both the intervention and comparison groups. Knowledge scores will be calculated by summing correct responses to generate a composite score, with higher scores indicating better disease‐related knowledge.

##### 2.10.2.3 Retention in Care

Retention in care will be assessed over the same 6‐month follow‐up period in both groups and measured based on participants' attendance at scheduled clinic visits and continuity of follow‐up care. This outcome will be used to evaluate ongoing engagement with HIV‐HBV care services.

#### 2.10.3 Exploratory Outcomes

Exploratory analyzes will be conducted to identify sociodemographic, clinical, and behavioral factors associated with virological response. In addition, the relationships among changes in HIV–HBV‐related knowledge, medication adherence, retention in care, and virological outcomes will be examined using correlation and multivariable regression analyzes. All exploratory variables will be collected during the same follow‐up period and assessed at equivalent time points across both study groups.

### 2.11 Intervention Implementation

**Figure 1:**
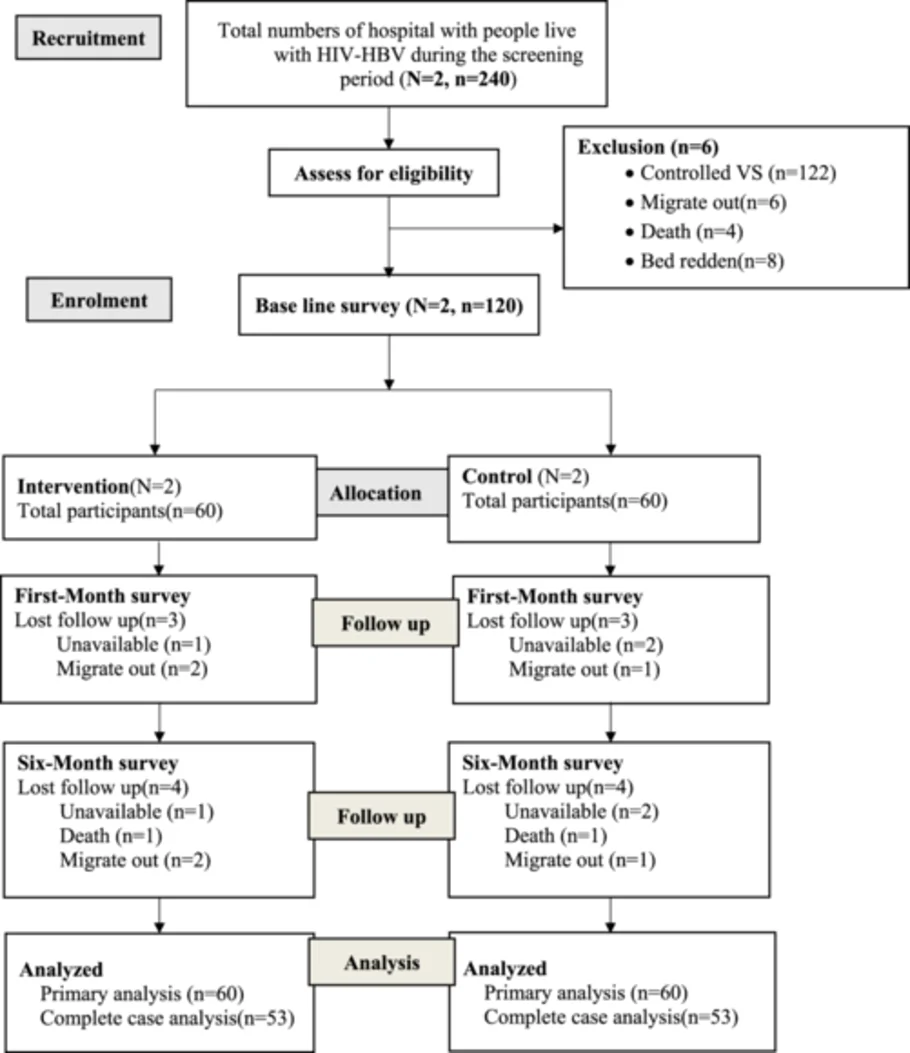
The CONSORT flow diagram of participant allocation, follow up, and analysis of effect of patient‐centered education on HIV virological suppression among unsuppressed adult people living with HIV‐HBV co‐infection in North‐West Ethiopia (*n* = 120).

Two hospitals with established HIV ART follow‐up services and pre‐identified eligible adults living with HIV will be included in the study. Because this is a pilot quasi‐experimental study, the hospitals will be purposively assigned to either the intervention or comparison group based on service capacity, logistical feasibility, and the need to minimize contamination between study arms. One hospital will serve as the intervention site, where 60 participants will receive the patient‐centered education intervention in addition to routine ART care, while the other hospital will serve as the comparison site, where 60 participants will continue receiving standard HIV care. Thus, a total of 120 adults living with HIV will be enrolled in the study, with 60 participants in the intervention group and 60 participants in the comparison group (Figure 1).

#### 2.11.1 Health Education Model for HIV VS

A framework for comprehending social behavior and personal cognition is provided by the Health Belief Model. Social psychologists first proposed it in the 1950s to elucidate the motivations underlying people's participation in health programs, such as vaccinations and physical examinations [25]. The Health Belief Model provides a framework for understanding social behavior and personal cognition, or an illness, and acceptance of particular health recommendations all of which are essential for improving health and reducing the difficulties of disease all influence their health‐related behaviors [26].

#### 2.11.2 Components of the Health Education Model

The Health Belief Model (HBM) is a widely recognized theoretical framework used to explain health‐related behaviors by examining how individuals perceive health risks and respond to recommended preventive or treatment‐related actions. The model provides a useful framework for understanding social and cognitive factors that influence health behaviors and for designing interventions aimed at improving health outcomes and reducing disease‐related complications. In this study, the HBM serves as the conceptual framework for the patient‐centered education intervention.

**Figure 2:**
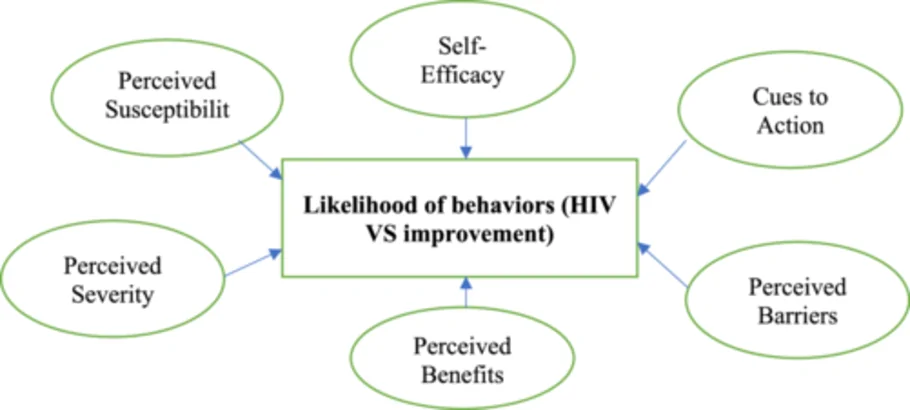
Inter‐relationship between variables of the health belief model, which will be used to explain HIV‐HBV co‐infected virological suppression improvement.

The HBM proposes that an individual's likelihood of engaging in a health‐promoting behavior is influenced by several key constructs. These include perceived susceptibility, which refers to an individual's subjective perception of their risk of developing a disease or experiencing complications; perceived severity, which reflects beliefs about the seriousness and potential consequences of the condition; perceived benefits, which describe beliefs regarding the effectiveness of recommended actions in reducing disease risk or improving health outcomes; and perceived barriers, which represent perceived obstacles or challenges that may hinder adoption of the recommended behavior. The model also includes cues to action, which are internal or external triggers that motivate behavior change, and self‐efficacy, which refers to an individual's confidence in their ability to successfully perform the recommended health behaviors. Together, these constructs provide a framework for understanding and improving behaviors related to medication adherence, retention in care, and self‐management among adults living with HIV–HBV co‐infection (Figure 2).

#### 2.11.3 Procedure of the Health Education Intervention

After obtaining written informed consent, participants in the intervention group will complete a structured interviewer‐administered questionnaire to collect baseline data on socio‐demographic characteristics, clinical status, HIV‐HBV‐related knowledge, Health Belief Model (HBM) constructs, medication adherence, self‐management practices, and retention in care. Baseline clinical data, including HIV viral load, ART regimen, and relevant laboratory findings, will also be extracted from participants' medical records and laboratory reports.

Following baseline assessment, the patient‐centered health education intervention will be delivered to participants in the intervention group by trained healthcare professionals (HCPs), including nurses, pharmacists, and clinicians. The intervention will be conducted through face‐to‐face educational sessions in Amharic using standardized educational materials, PowerPoint presentations, interactive discussions, counseling sessions, and audiovisual teaching aids designed to enhance participant engagement and understanding.

The intervention will be delivered during routine ART follow‐up visits over a 6‐month period. Participants in the intervention group will receive structured monthly education and counseling sessions, with each session lasting approximately 40–60 min. Sessions will focus on HIV–HBV co‐infection, disease transmission, medication adherence, viral load monitoring, stigma reduction, self‐management practices, and behavioral strategies to improve retention in care. Family members or treatment supporters may participate in selected sessions, where appropriate, to strengthen social support and adherence.

Six trained healthcare professionals will implement the intervention at the designated intervention hospital. The intervention package, including educational content and delivery procedures, will be reviewed and validated by subject matter experts to ensure content accuracy, clarity, and consistency. To ensure intervention fidelity, all participants in the intervention group will receive the same educational content, dosage, frequency, and duration throughout the study period. Attendance will be documented, and participants who miss scheduled sessions will be contacted and rescheduled whenever feasible.

Outcome assessments will be conducted at two time points: baseline and at the end of the 6‐month follow‐up period. Changes in primary and secondary outcomes including HIV virological outcomes, medication adherence, HIV‐HBV‐related knowledge, self‐management practices, and retention in care will be compared between the intervention and comparison groups to evaluate the effectiveness of the patient‐centered education intervention.

### 2.12 Data Collection Procedures

#### 2.12.1 Baseline Survey

A baseline survey will be conducted prior to implementation of the intervention. Data will be collected through participant interviews and abstraction of clinical, laboratory, and patient medical records. Trained study nurses or research assistants will interview adult participants living with HIV–HBV co‐infection during enrollment using a structured questionnaire to collect socio‐demographic, clinical, behavioral, and HIV/HBV‐related information. Clinical and laboratory data, including HIV viral load, ART regimen, CD4 count, and other relevant medical information, will be extracted from participants' medical records and laboratory reports. In addition, Health Belief Model (HBM) constructs including perceived susceptibility, perceived severity, perceived benefits, perceived barriers, cues to action, and self‐efficacy as well as medication adherence and baseline knowledge regarding HIV–HBV co‐infection, will be assessed during the baseline evaluation.

#### 2.12.2 Follow‐Up and Endline Survey

Data will be collected at two time points: baseline (enrollment) and at the end of the 6‐month follow‐up period. At both assessment points, trained research nurses will collect socio‐demographic, clinical, and behavioral information through participant interviews and abstraction of medical records. Information on clinic attendance, retention in care, and pharmacy medication refill history among participants receiving ART will also be obtained from patient medical records. HIV viral load data will be collected at baseline and at the scheduled 6‐month follow‐up visit using laboratory viral load testing reports or dried blood spot (DBS) samples, where applicable. In addition, data on medication adherence, Health Belief Model (HBM) constructs, self‐management practices, and HIV–HBV‐related knowledge will be collected using structured questionnaires. All primary, secondary, and exploratory outcome measures will be assessed using data collected at these two time points to enable comparison of changes over time between the intervention and comparison groups.

##### 2.12.2.1 Patient Demographics and Treatment Variables

A structured data abstraction form and interviewer‐administered questionnaire will be used to collect participants' socio‐demographic, clinical, behavioral, and treatment‐related information under the supervision of the research team, which includes the principal investigator, a statistician, a clinical pharmacist, and senior medical experts. Trained research nurses will collect the data, while the principal investigator will oversee data quality through regular monitoring and validation.

Variables to be collected include age, sex, duration since HIV diagnosis, duration on ART, baseline and follow‐up HIV viral load, CD4 count, HBV serological status, comorbidities, concurrent medications, medication adherence, Health Belief Model (HBM) constructs, retention in care, and HIV–HBV‐related knowledge scores. These variables will be recorded at baseline and reassessed at the 6‐month follow‐up to evaluate changes over time and estimate the effect of the intervention.

Data will be obtained from participant interviews, patient medical records, laboratory reports, pharmacy refill records, and health facility electronic databases where available. All collected data will be anonymized by assigning unique study identification numbers before analysis to ensure participant confidentiality. Electronic datasets will be securely stored on password‐protected computers accessible only to authorized members of the research team.

Ethical approval will be obtained from the Institutional Review Board of the University of Gondar. Written informed consent will be obtained from all participants before enrollment. Permission to use standardized questionnaires and assessment tools will be secured from the respective developers or authorized sources prior to data collection.

#### 2.12.3 The Health Belief Model Questionnaires

Data on participants' knowledge regarding HIV–HBV co‐infection and Health Belief Model (HBM) constructs will be collected at baseline (enrollment) and at the end of the 6‐month follow‐up period using a structured interviewer‐administered questionnaire. The questionnaire will assess knowledge (15 items) and the six core HBM domains, including perceived susceptibility (8 items), perceived severity (7 items), perceived benefits (8 items), perceived barriers (7 items), self‐efficacy (8 items), and cues to action (6 items).

Responses to HBM items will be measured using a 5‐point Likert scale ranging from 1 (strongly disagree) to 5 (strongly agree), reflecting participants' perceptions, beliefs, motivation, and confidence regarding adherence to recommended HIV–HBV‐related health behaviors, including medication adherence, clinic attendance, and self‐management practices. Knowledge items will be scored based on correct responses, and a composite knowledge score will be generated, with higher scores indicating better knowledge. Composite scores will also be calculated for each HBM construct by summing item responses, with higher scores indicating stronger beliefs within the respective domains. Changes in knowledge and HBM construct scores between baseline and the 6‐month follow‐up assessment will be evaluated to determine the effect of the patient‐centered education intervention [27].

#### 2.12.4 Virological Suppression

HIV viral load measured at baseline and at the end of the 6‐month follow‐up period will be used to assess virological outcomes. The primary virological outcome will be evaluated as the change in log‐transformed HIV viral load (log_10_ copies/mL) between baseline and follow‐up. In addition, virological suppression will be assessed as a secondary clinical outcome and will be defined as an HIV viral load of < 1000 copies/mL at the 6‐month follow‐up assessment, consistent with recommended clinical thresholds for treatment success. HIV RNA levels will be measured using a real‐time polymerase chain reaction (PCR) assay. Blood samples or routine laboratory viral load testing reports will be obtained through referral hospital laboratories accredited for HIV viral load testing. Additional clinical information related to virological outcomes will also be extracted from participants' medical records and laboratory reports.

#### 2.12.5 Medication Adherence

Medication adherence will be assessed at baseline and at the end of the 6‐month follow‐up period using a context‐adapted version of the Hill‐Bone Medication Adherence Scale (HBMAS), a widely used, simple, and reliable self‐report instrument for evaluating adherence behaviors in chronic disease management. For this study, the scale has been adapted to assess adherence to ART and HBV‐active medications among adults living with HIV‐HBV co‐infection.

The adapted HBMAS evaluates three key adherence domains: medication‐taking behavior, appointment keeping, and prescription refilling, while maintaining the original conceptual framework of the instrument. Responses will be recorded using a Likert‐type scale, and item scores will be summed to generate a total adherence score, with higher total scores indicating poorer medication adherence.

Medication adherence will primarily be analyzed as a continuous outcome using the total HBMAS score to assess changes in adherence between baseline and the 6‐month follow‐up period. Mean changes in adherence scores between the intervention and comparison groups will be evaluated to estimate the effect of the patient‐centered education intervention. Additionally, adherence will be categorized as good or poor based on predefined cut‐off values derived from the total HBMAS score for supplementary categorical analyzes. Associations between the intervention and adherence status at follow‐up will be explored using appropriate regression models while adjusting for relevant baseline covariates. The adapted HBMAS has been widely used in clinical and research settings and is considered an appropriate tool for identifying varying levels of medication adherence in this study population [23, 24].

### 2.13 Variable Measurement and Definition

#### 2.13.1 Virological Suppression (HIV)

Virological suppression will be defined as an HIV viral load of < 1000 copies/mL of plasma, or below the laboratory assay detection limit where applicable, based on the most recent viral load measurement. This definition is consistent with the World Health Organization (WHO) criteria for monitoring ART response and treatment success [28].

#### 2.13.2 Medication Adherence

a secondary outcome variable, will be measured using a context‐adapted version of the Hill‐Bone Medication Adherence Scale (HBMAS). The HBMAS is a validated self‐report instrument originally developed to assess medication‐taking behaviors in chronic disease management and has been widely used in both clinical practice and research settings. For the present study, the scale has been adapted to assess adherence to ART and HBV‐active medications among adults living with HIV–HBV co‐infection. Item wording has been modified to reflect ART dosing schedules, routine HIV clinic attendance, and prescription refilling practices while preserving the original conceptual domains of medication‐taking behavior, appointment keeping, and prescription refilling.

Responses to the adapted HBMAS will be recorded using a Likert‐type response scale, with higher scores indicating poorer medication adherence. A composite adherence score will be calculated by summing individual item responses. Medication adherence will primarily be analyzed as a continuous outcome using the total adherence score to evaluate changes between baseline and the 6‐month follow‐up period. For supplementary analyzes, adherence will also be categorized into predefined adherence levels (e.g., good vs. poor adherence), with lower scores representing better adherence and higher scores indicating suboptimal adherence.

The adapted scale will undergo translation and back‐translation procedures to ensure linguistic and conceptual equivalence. Internal consistency reliability will be assessed using Cronbach's alpha prior to analysis. The context‐adapted HBMAS is considered an appropriate and reliable tool for assessing medication adherence among adults living with HIV in the study setting [23, 24].

### 2.14 Qualitative Data

A qualitative study will be conducted at the end of the intervention period to explore the acceptability, feasibility, and perceived usefulness of the patient‐centered health education intervention among adults living with HIV receiving ART, healthcare providers, and hospital leaders in North‐West Ethiopia. A sequential interpretive descriptive qualitative approach will be employed to obtain an in‐depth understanding of participants' experiences, perceptions, and contextual factors influencing the implementation of the intervention.

Data will be collected using three qualitative methods: focus group discussions (FGDs) with adults living with HIV–HBV co‐infection, in‐depth interviews (IDIs) with healthcare providers involved in HIV/HBV care, and key informant interviews (KIIs) with hospital leaders and program managers. These methods will enable exploration of patient experiences, provider perspectives, and institutional‐level barriers and facilitators related to implementation and sustainability of the intervention.

Data collection will be guided by pretested semi‐structured interview guides developed by the research team based on relevant literature and study objectives. Separate interview guides will be prepared for FGDs, IDIs, and KIIs to ensure context‐specific exploration of issues. The guides will initially be developed in English, translated into Amharic, and back‐translated to ensure linguistic consistency and conceptual equivalence. Necessary revisions will be made following pretesting to improve clarity and contextual relevance.

Face‐to‐face interviews and focus group discussions will be conducted using open‐ended questions supported by probing and follow‐up questions to facilitate deeper exploration of emerging themes. All FGDs, IDIs, and KIIs will be audio‐recorded using electronic devices (smartphones), and field notes will be taken to capture contextual observations and non‐verbal cues. Data collection will take place in private rooms within the outpatient departments (OPDs) or administrative offices of participating hospitals to ensure privacy and minimize interruptions.

The FGDs will be facilitated by experienced moderators fluent in the local language and assisted by trained note‐takers familiar with the cultural context. The principal investigator will supervise all qualitative data collection activities to ensure consistency and data quality. Each IDI and KII is expected to last approximately 40–60 min, while each FGD will last approximately 60–90 min.

Participation in the qualitative study will be voluntary, and written informed consent will be obtained from all participants prior to data collection. Data collection will continue until data saturation is achieved, defined as the point at which no new themes or meaningful insights emerge from additional interviews or discussions.

### 2.15 Data Analysis

Data will enter into EpiData version 4.6 and exported to Stata version 18.0 (StataCorp, College Station, TX, USA) for analysis. Data cleaning, coding, and consistency checks will be performed before statistical analysis.

Descriptive statistics will used to summarize participants' socio‐demographic, clinical, behavioral, and treatment‐related characteristics at baseline. Continuous variables will presented as mean ± standard deviation (SD) for normally distributed data or median with interquartile range (IQR) for skewed distributions, whereas categorical variables will be summarized using frequencies and percentages. Baseline characteristics between the intervention and comparison groups were compared using the independent t‐test or Mann‐Whitney U test for continuous variables and the Chi‐square or Fisher's exact test for categorical variables, as appropriate.

The primary outcome is HIV viral load, analyzed as a continuous variable. Secondary outcomes included medication adherence and HIV‐related knowledge scores, both treated as continuous variables. Medication adherence will be assessed using the adapted Hill‐Bone Medication Adherence Scale (HBMAS), with lower scores indicating better adherence, while HIV‐related knowledge was measured using a structured questionnaire, with higher scores indicating better knowledge.

Within‐group changes from baseline to 6 months were assessed using paired t‐tests for normally distributed continuous variables. The intervention effect will be evaluated using the Difference‐in‐Differences (DiD) approach, calculated as: DiD = $Y_{I,\text{pre}}$represent the mean outcome values for the intervention group at endline and baseline, respectively, and $Y_{C,\text{post}}$and $Y_{C,\text{pre}}$represent the corresponding values for the comparison group.

To estimate the adjusted intervention effect while accounting for repeated measurements within individuals and potential confounding, mixed‐effects linear regression models with random intercepts for participants will be fitted for each outcome. Fixed effects included group (intervention vs. comparison), time (baseline vs. endline), and the group‐by‐time interaction, with the interaction term representing the DiD estimate of the intervention effect. Models will adjust for clinically relevant covariates, including age, sex, religion, marital status, educational level, residence, WHO clinical stage, opportunistic infections, treatment interruption, alcohol use, smoking status, and condom use. Adjusted beta (β) coefficients with their corresponding 95% confidence intervals (CIs) were reported.

Model assumptions, including normality of residuals, linearity, homoscedasticity, independence of observations, and multicollinearity, were evaluated before model interpretation. Random‐effects assumptions will be assessed using the estimated variance components and intraclass correlation coefficient (ICC). All statistical tests were two‐sided, and statistical significance will be declared at *p* < 0.05.

Because this is a pilot quasi‐experimental study with a relatively small sample size, greater emphasis will placed on the magnitude and precision of the estimated intervention effects, expressed as adjusted β coefficients and 95% confidence intervals, rather than statistical significance alone. These findings are intended to inform the design and sample size estimation of future adequately powered randomized or multicenter intervention studies.

## 3 Discussion

Due to the high endemicity of both illnesses and their combined clinical impact, HIV continues to be a major public health concern throughout sub‐Saharan Africa, especially in Ethiopia. Health care professionals' use of patient‐centered education interventions has demonstrated a favorable outcome in terms of achieving virological suppression, better medication adherence, and optimal viral load control. Patient‐centered education programs have been successful in controlling infectious illnesses and enhancing the health of mothers and children in Ethiopia. Even when treated with ART regimens active against HBV (such as tenofovir‐based combinations), co‐infected patients are more likely to experience unfavorable clinical outcomes, such as slower immunological recovery, increased liver‐related complications, and decreased likelihood of virological suppression [29, 30]. These difficulties highlight the need for creative approaches to improve ART efficacy in this population that go beyond medication.

A potential but underutilized strategy for removing obstacles to the best possible HIV outcomes is patient‐centered education [31]. Research indicates that customized education raises awareness of comorbid diseases, improves ART adherence, and increases patient involvement in care, all of which lead to improved virological responses [32, 33]. However, the majority of current initiatives target individuals with HIV mono‐infection and seldom concentrate on comorbidity situations, such as HIV‐HBV co‐infection, where patient counseling and information gaps can be even more important because managing dual diseases can be challenging [34]. Therefore, by assessing the impact of a planned, patient‐centered education program designed especially for people co‐infected with HIV and HBV, this study is positioned to close a significant gap.

Particularly in disadvantaged groups getting routine care, the quasi‐experimental methodology chosen for this study offers a practical and moral substitute for randomized controlled trials [35]. It permits the educational intervention to be implemented in the actual world while allowing comparison with a control group receiving routine care. This strategy improves external validity, particularly for findings that are relevant to policy, and boosts feasibility in places with limited resources. However, through matching, statistical adjustment, and standardized delivery of intervention content, inherent constraints such as the absence of randomization and possible selection bias will be lessened. The results may provide compelling evidence for incorporating patient‐centered education into HIV‐HBV treatment recommendations in Ethiopia and other contexts if the intervention shows enhanced HIV viral suppression [36]. The results may help develop more sustainable ways to support the UNAIDS 95‐95‐95 targets because educational activities are inexpensive and scalable across ART facilities [37]. Additionally, this study might encourage future investigation into patient‐centered strategies for treating chronic illnesses and other co‐infections in HIV programs. Overall, this approach shows how structured education can empower patients to improve clinical outcomes beyond medication‐based interventions, especially in situations where co‐infection presents extra treatment obstacles. Patient‐centered approaches may be crucial for improving treatment results in Ethiopia and other high‐burden areas as HIV continues to put a burden on already restricted healthcare systems.

This study has several methodological limitations. The intervention and control groups are recruited from only two health facilities, with one facility serving as the intervention site and the other as the control site. As a result, differences in outcomes may reflect not only the effect of the intervention but also underlying site‐specific characteristics, making it challenging to fully separate intervention effects from facility‐level influences. In addition, the inclusion of only two sites precludes the use of clustering‐adjusted analytical approaches such as mixed‐effects models or generalized estimating equations. Although multivariable analyzes will be performed to adjust for measured baseline differences between groups, residual confounding arising from unmeasured participant‐ or facility‐level factors may remain. Therefore, the study findings should be interpreted cautiously, taking into account the potential impact of unmeasured site‐specific influences.

## 4 Limitations of the Study

As this is a pilot quasi‐experimental study with a relatively small sample size, the study may have limited statistical power to detect statistically significant differences in HIV virological outcomes between the intervention and comparison groups. Therefore, the findings should be interpreted cautiously, as the study is primarily intended to evaluate feasibility, acceptability, and generate preliminary estimates of intervention effects to inform the design and sample size calculation of future adequately powered studies. In addition, baseline HIV drug resistance testing, systematic TB screening, and longitudinal ALT monitoring were not performed because of resource and laboratory constraints. Consequently, adherence‐related virological failure could not be distinguished from resistance‐related failure, and liver safety outcomes could not be evaluated. Furthermore, validated measures of HIV‐related stigma and depression were not included, which may have limited the assessment of important behavioral and psychosocial factors influencing medication adherence and virological outcomes. These factors should be considered in future studies.

## 5 Trial Status Timeline of the Study

The study will be conducted until completion of the end‐line data collection and final analysis. Participant recruitment and baseline assessment will commence in December 2025. The patient‐centered education intervention and follow‐up data collection will be conducted from January to June 2026, with end‐line assessments completed at the end of the 6‐month follow‐up period. Data cleaning, analysis, and interpretation will be carried out thereafter, and the final study results are expected by July 2026. A total of 120 adults living with HIV receiving ART will be enrolled from two specialized referral hospitals, with 60 participants in the intervention group and 60 participants in the comparison group.

## Author Contributions

**Mequanente Dagnaw:** conceptualization, funding acquisition, investigation, writing – original draft, methodology, visualization, formal analysis, data curation. **Destaw Fetene Teshome:** investigation, validation, software, resources. **Tilahun Bizuayehu Demass:** investigation, methodology, software, data curation. **Abebaw Gebyehu Worku:** conceptualization, investigation, writing – original draft, writing – review and editing, supervision.

## Funding

The authors have nothing to report.

## Ethics Statement

The College of Medicine and Health Sciences and Specialized Hospital (CMHS & SH) Institutional Research Ethics Review Committee (IRERC) approved the study (Ref. No. CMHSSH‐UoGIRERC/108/11/2025; November 12, 2025). Written informed consent was obtained from each participant using a signature or thumbprint after providing detailed information about the study objectives, procedures, potential risks, and benefits. Participants in the intervention group may benefit from improved HIV‐related knowledge, medication adherence, self‐management practices, and engagement in HIV care. To ensure privacy, interviews and educational sessions were conducted in a private setting at the ART clinic. Confidentiality was maintained by using unique identification codes instead of participants' names, securely storing study records, and restricting data access to authorized research personnel. Participation was entirely voluntary, and participants could decline or withdraw at any time without affecting their routine HIV care. The patient‐centered education was integrated into participants' routine ART follow‐up visits to minimize additional time and travel burden. No financial compensation was provided for participation, as no separate 3‐h study visits were required.

## Conflicts of Interest

The authors declare no conflicts of interest.

## Transparency Statement

The lead author Mequanente Dagnaw affirms that this manuscript is an honest, accurate, and transparent account of the study being reported; that no important aspects of the study have been omitted; and that any discrepancies from the study as planned (and, if relevant, registered) have been explained.

## Data Availability

The data that support the findings of this study are available from the corresponding author upon reasonable request. Because HIV‐related information is sensitive and participant privacy and confidentiality must be protected, the qualitative data created and evaluated during the current study are not publicly available. The corresponding author may make de‐identified data available upon reasonable request and with the necessary ethical permissions from the relevant institutional review boards.
